# Hsa_circ_0051908 Promotes Hepatocellular Carcinoma Progression by Regulating the Epithelial–Mesenchymal Transition Process

**DOI:** 10.1155/2024/8645534

**Published:** 2024-04-30

**Authors:** Yinbing Wu, Huafei Tang, Shuzhong Cui, Quanxing Liao, Lisi Zeng, Yinuo Tu

**Affiliations:** ^1^Department of Hepatobiliary Surgery, Affiliated Cancer Hospital and Institute of Guangzhou Medical University, Guangzhou, China; ^2^Affiliated Cancer Hospital and Institute of Guangzhou Medical University, Guangzhou, China

## Abstract

**Materials and Methods:**

Hsa_circ_0051908 expression was determined using RT-qPCR. HCC cell proliferation, apoptosis, invasion, and migration were assessed using CCK-8 assay, EdU staining, TUNEL staining, flow cytometry, and transwell assay. The molecular mechanism was analyzed using western blotting. In addition, the role of hsa_circ_0051908 in tumor growth was evaluated *in vivo*.

**Results:**

Hsa_circ_0051908 expression was increased in both HCC tissues and cell lines. The proliferation, migration, and invasion of HCC cells were significantly decreased after hsa_circ_0051908 knockdown, while cell apoptosis was notably increased. Furthermore, we found that hsa_circ_0051908 silencing downregulated vimentin and Snail and upregulated E-cadherin. *In vivo*, hsa_circ_0051908 silencing significantly inhibited the growth of the tumor.

**Conclusions:**

Our data provide evidence that hsa_circ_0051908 promotes HCC progression partially by mediating the epithelial–mesenchymal transition process, and it may be used for HCC treatment.

## 1. Introduction

Hepatocellular carcinoma (HCC) is a highly heterogeneous malignancy that causes death [1]. By 2025, more than one million cases of HCC may have been diagnosed globally [2]. Although the diagnosis and treatment of HCC have improved, some patients with HCC lose the opportunity for surgery owing to late detection [1–3]. Moreover, the recurrence rate of HCC metastasis is high [4, 5]; therefore, the identification of new biomarkers and therapeutic targets for HCC treatment is warranted.

CircRNAs are a class of RNAs with a relatively stable structure formed by a special loop formation mechanism. They exist widely in eukaryotic cells and regulate tumor development and immune responses [6, 7]. Several studies indicated that circRNAs regulate the occurrence and malignant progression of HCC [8, 9]. Wei et al. [10] found that circ-CDYL promotes HCC progression by mediating the noncoding RNA regulatory network and activating the PI3K/AKT and HIF1AN/Notch2 signaling pathways. Liang et al. [11] found that circ*β*-catenin competitively binds to GSK3*β* by encoding a short peptide and acts as bait for GSK3*β*, thus enhancing the stability of *β*-catenin, promoting its entry into the nucleus, and activating the Wnt/*β*-catenin pathway. Additionally, a study showed that circUHRF1 can reduce the therapeutic effect of anti-PD-1 in patients with HCC by targeting miR-449c-5p [12]. Owing to their unique biological characteristics, stability of closed-loop structure, and differential expression in tissues and body fluids, circRNAs have a wide application prospect in the early diagnosis and treatment of HCC. Furthermore, microarray results showed high expression of hsa_circ_0051908 in liver cancer tissues, indicating that it might play an important role in the development of liver cancer. However, its role in HCC development is still unknown. Therefore, hsa_circ_0051908 was selected for further evaluation.

Epithelial–mesenchymal transition (EMT) is a biological process in which epithelial cells, which are polarized and organized into layers, lose their characteristics, and acquire mesenchymal phenotypes that are associated with increased motility and invasiveness. EMT promotes cancer cells to detach from the primary tumor, invade surrounding tissues, and metastasize to distant organs [13]. The EMT process in HCC involves the activation of several signaling pathways, including the transforming growth factor-*β* (TGF-*β*), Wnt/*β*-catenin, and notch pathways [14]. These pathways regulate the expression of EMT-associated transcription factors, such as Snail, Slug, Twist, and ZEB1/ZEB2, which bind to the promoters of genes involved in cell adhesion, cytoskeleton remodeling, and extracellular matrix degradation [15]. In addition, the EMT process is associated with poor prognosis and resistance to chemotherapy and immunotherapy in HCC [16]. Therefore, targeting EMT and its associated pathways via hsa_circ_0051908 may be a novel approach to treat HCC.

In this study, we explored the expression of hsa_circ_0051908 in HCC tumors and cell lines and examined the effects of hsa_circ_0051908 silencing on the biological behaviors of HCC cells. Our study findings suggest that hsa_circ_0051908 may serve as a potential therapeutic target for HCC by regulating the EMT process.

## 2. Materials and Methods

### 2.1. Tissue Specimen Collection

HCC tissues were collected from 70 patients with HCC at Affiliated Cancer Hospital and Institute of Guangzhou Medical University, and experiments using clinical specimens were approved by the ethics committee of Affiliated Cancer Hospital and Institute of Guangzhou Medical University. The inclusion criteria were as follows: (i) HCC diagnosed by radiology, histology, or the International Classification of Disease (ICD) criteria; (ii) participants without any subtype of cancer at the beginning of the study; (iii) age > 18 years. The exclusion criteria were as follows: (i) receiving other antitumor treatments; (ii) lost to follow-up; and (iii) incomplete clinical data.

### 2.2. Cell Culture

Human hepatocytes (WRL68 cells) and HCC cells (HepG2, SK-Hep-1, Huh7, and SMMC772) were obtained from the American Type Culture Collection (Manassas, VA, USA). WRL68, SK-Hep-1, and HepG2 cells were cultured in minimum essential medium (Procell, Wuhan, China, PM150410) supplemented with 10% fetal bovine serum (FBS, HyClone, USA, SH30396), while SMMC7721 and Huh7 cells were, respectively, cultured in RPMI-1640 medium and DMEM (Procell, PM150110B) supplemented with 10% FBS.

### 2.3. Reverse Transcription (RT)-PCR

The TRIzol kit (Takara, Dalian, China, 9108Q) was used to extract total RNA from the samples and confirm the integrity and concentration of total RNA before PCR amplification. cDNA was synthesized using the Bestar qPCR RT Kit (DBI Bioscience, Germany, DBI-2220). Real-time PCR was run on the Agilent Stratagene Mx3000P PCR system (Agilent, CA, USA, Mx3000P) using Bestar® SYBR Green qPCR master Mix (DBI Bioscience, DBI2044). The relative expression of hsa_circ_0051908 was calculated using the 2^−*ΔΔ*Ct^ method with GAPDH as the internal control. The following primers were used: hsa_circ_0051908 (F: 5′-ATT CCA CTG AGC GTG CCT AC-3′; R: 5′-AAT GTA GGT GCC CTC AAT AGC-3′) and GAPDH (F: 5′-TGT TCG TCA TGG GTG TGA AC-3′; R: 5′-ATG GCA TGG ACT GTG GTC AT-3′).

### 2.4. RNA Interference and Transfection

HCC cells were transfected with hsa_circ_0051908 siRNA and negative control (NC) siRNA [2] using Lipofectamine RNAi MAX (Invitrogen, CA, USA, 13778150) to inhibit hsa_circ_0051908 expression. Hsa_circ_0051908 siRNA was synthesized by RuiboBio (Guangzhou, China).

### 2.5. CCK-8 Assay

Briefly, 2 × 10^3^ transfected HCC cells (HepG2 and Huh7) were seeded in 96-well plates. After culturing for indicated times, the CCK-8 solution was added and the absorbance value at 450 nm was measured.

### 2.6. EdU Staining

The transfected HCC cells (1 × 10^5^ cells/well) were cultured in 12-well plates. After the indicated time, the medium was replaced with a medium containing EdU (50 *μ*M, Beyotime, Nanjing, China, C0078S) and then cultured for 2 hr. Nuclei were stained with 4,6-diamino-2-phenyl indole (DAPI, Solarbio, Beijing, China, DA0004). Images were captured using a fluorescence microscope.

### 2.7. Apoptosis Assay

HCC cells (5 × 10^5^) were collected after centrifugation at 1,000 rpm and 4°C for 5 min. Then, 5 *μ*L Annexin V-FITC (Solarbio, CA1020) and 5 *μ*L propidium iodide staining solution were added for staining. The samples were analyzed using flow cytometry.

### 2.8. TUNEL Staining

HCC cells were transfected with hsa_circ_0051908 and NC siRNA. After 48 hr, HCC cells were fixed and the cell membrane was permeated with 4% paraformaldehyde and 0.1% Triton X-100 solution, respectively. The cells were incubated with 50 *μ*L TUNEL solution (Beyotime, C1089), and then nuclei were stained with DAPI, followed by detection using a fluorescence microscope.

### 2.9. Cell Migration and Invasion Assays

For the migration assay, 2 × 10^4^ transfected HCC cells were seeded into the upper transwell chamber (Corning, 3422). The complete medium was added to the 24-well plate (outer chamber). After 48 hr, the fixed cells were stained with 0.4% crystal violet. The migrated cells were observed using a microscope.

For the invasion assay, matrigel (BD, 356234) was polymerized in transwell chambers at 37°C for 60 min. Then, 4 × 10^4^ transfected HCC cells were seeded on polymerized matrigel. The subsequent experimental steps were the same as those of the cell migration assay.

### 2.10. Western Blotting

The same amounts of protein samples were separated using sodium dodecyl sulfate-polyacrylamide gel electrophoresis and transferred onto PVDF membranes (Millipore, CA, USA, No. IPVH0010). The membranes were incubated with primary and secondary antibodies. Then, the ECL-Plus kit was used to visualize the blots. The antibodies used were as follows: E-cadherin (3195T, CST, USA), Snail (3879, CST), vimentin (5741, CST), and GAPDH (5174, CST).

### 2.11. Animal Models

Ten male BALB/c nude mice received a subcutaneous injection of 100 *μ*L HepG2 cells (1 × 10^6^ cells). When the tumor volume was approximately 50 mm^3^, the nude mice were randomly divided into two groups (*N* = 5): NC and siRNA groups. Mice in the NC group were intratumorally injected with 10 nmol NC siRNA and those in the siRNA group were intratumorally injected with 10 nmol hsa_circ_0051908 siRNA. Mice were injected once every 3 days for four times in total. Four weeks after siRNA injections, all mice were euthanized.

### 2.12. Statistical Analysis

One-way ANOVA and Student's *t*-test were used to analyze differences among and between the groups, respectively. Data are expressed as mean ± standard deviation (SD). Statistical analyses were performed using GraphPad Prism 8.0 software (GraphPad, CA, USA). A *P* value < 0.05 was considered statistically significant.

## 3. Results

### 3.1. Hsa_circ_0051908 Was Upregulated in HCC

**Figure 1:**
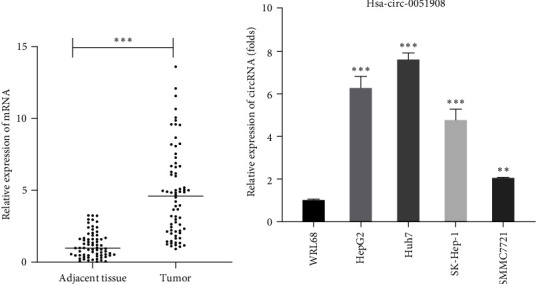
Hsa_circ_0051908 expression in HCC tissues and cells. (a, b) Hsa_circ_0051908 expression in HCC tissues, matched adjacent tissues, and HCC cells were detected using RT-qPCR. All experiments were replicated three times.  ^*∗∗*^*P*  < 0.01;  ^*∗∗∗*^*P*  < 0.001.

Microarray results have shown that hsa_circ_0051908 is highly expressed in liver cancer tissues [17]. Here, we found that hsa_circ_0051908 expression was higher in HCC tissues (*N* = 70) than in adjacent tissues (Figure 1(a)). Moreover, the expression of hsa_circ_0051908 in normal human hepatocytes (WRL68 cells) and HCC cells (HepG2, Huh7, SK-Hep-1, and SMMC7721) was analyzed using RT-qPCR. The results showed that hsa_circ_0051908 expression was greatly higher in HCC cells, especially in HepG2 and Huh7 cells, than in WRL68 cells (Figure 1(b)). In addition, the expression of hsa_circ_0051908 was significantly correlated with tumor size, tumor differentiation, TNM stage, and lymph node metastasis (Table 1). These data suggest that hsa_circ_0051908 might play an important role in the progression of HCC.

### 3.2. Knockdown of hsa_circ_0051908 Suppressed the Proliferation of HCC Cells

**Figure 2:**
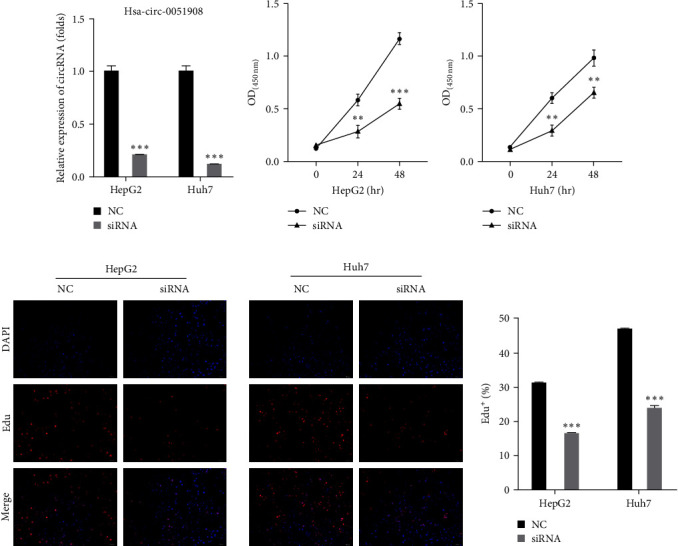
Hsa_circ_0051908 promoted the proliferation of HCC cells. (a) Successful transfection was confirmed using RT-qPCR. (b, c) The CCK-8 assay was performed to determine cell viability. (d, e) The proliferation of HCC cells was observed using EdU staining. All experiments were replicated three times.  ^*∗∗*^*P*  < 0.01;  ^*∗∗∗*^*P*  < 0.001.

Hsa_circ_0051908 siRNA was synthesized and transfected into HepG2 and Huh7 cells. RT-qPCR results showed that hsa_circ_0051908 siRNA effectively suppressed hsa_circ_0051908 expression in HepG2 and Huh7 cells (Figure 2(a)). In addition, we found that the viability of HepG2 and Huh7 cells transfected with hsa_circ_0051908 siRNA was obviously reduced at 24 and 48 hr (Figures 2(b) and 2(c)). Moreover, EdU staining demonstrated that hsa_circ_0051908 knockdown markedly suppressed the proliferation of HepG2 and Huh7 cells (Figures 2(d) and 2(e)).

### 3.3. Knockdown of hsa_circ_0051908 Increased Apoptosis of HCC Cells

**Figure 3:**
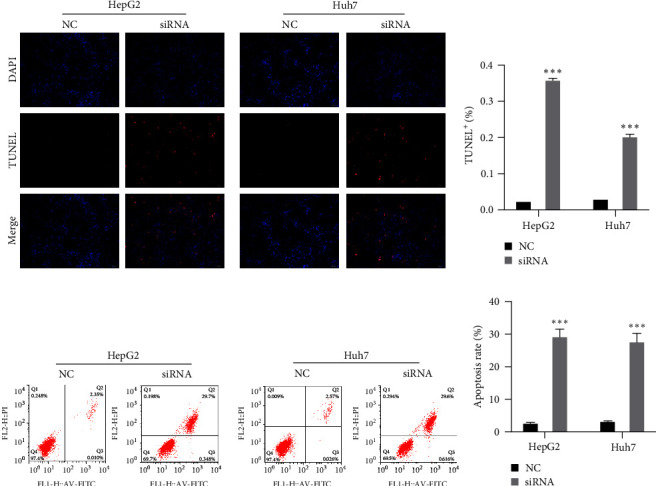
Hsa_circ_0051908 inhibited apoptosis of HCC cells. Apoptosis of HepG2 and Huh7 cells was evaluated using TUNEL staining (a, b) and flow cytometry assay (c, d). All experiments were replicated three times.  ^*∗∗∗*^*P*  < 0.001.

TUNEL staining results revealed that apoptosis of HepG2 and Huh7 cells transfected with hsa_circ_0051908 siRNA was obviously increased compared with that of the NC group at 48 hr (Figures 3(a) and 3(b)). Consistently, flow cytometry also indicated that hsa_circ_0051908 knockdown increased apoptosis of HepG2 and Huh7 cells (Figures 3(c) and 3(d)).

### 3.4. Knockdown of hsa_circ_0051908 Suppressed HCC Cell Migration, Invasion, and EMT

**Figure 4:**
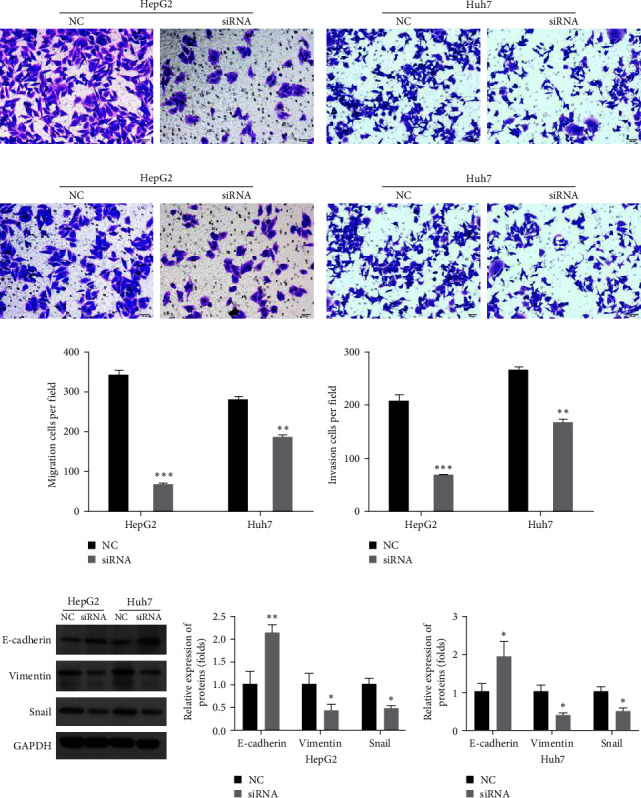
Hsa_circ_0051908 promoted HCC cell migration, invasion, and EMT. The migration (a, c) and invasion abilities (b, d) of HCC cells were observed using the migration and invasion assays, respectively. (e) Western blot analysis of the EMT markers in HCC cells. All experiments were replicated three times.  ^*∗*^*P*  < 0.05;  ^*∗∗*^*P*  < 0.01;  ^*∗∗∗*^*P*  < 0.001.

The migration ability of HCC cells transfected with hsa_circ_0051908 siRNA was notably suppressed at 48 hr (Figures 4(a) and 4(c)). Hsa_circ_0051908 knockdown also notably suppressed the invasion ability of HCC cells at 48 hr (Figures 4(b) and 4(d)). Moreover, hsa_circ_0051908 knockdown upregulated E-cadherin and downregulated vimentin and Snail in HCC cells (Figure 4(e)).

### 3.5. Knockdown of hsa_circ_0051908 Inhibited Tumor Growth In Vivo

**Figure 5:**
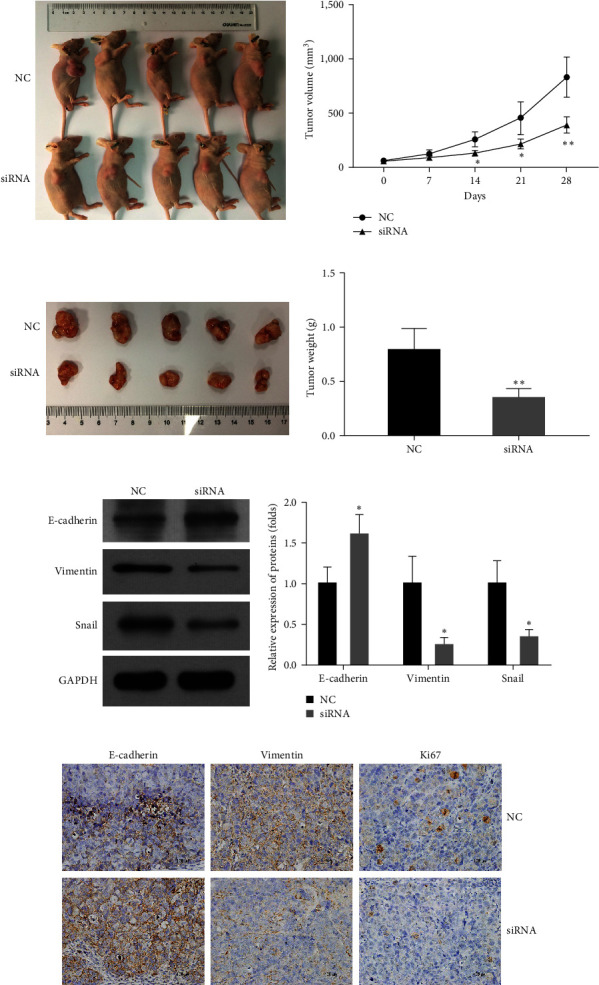
Hsa_circ_0051908 promoted tumor growth *in vivo*. Hsa_circ_0051908 knockdown decreased tumor volume (a) and weight (b) in BALB/c nude mice. (c) Western blot analysis of the EMT markers in xenograft tumors. (d) Immunohistochemical staining analysis of the EMT markers and Ki-67 in xenograft tumors. All experiments were replicated three times.  ^*∗*^*P*  < 0.05;  ^*∗∗*^*P*  < 0.01.

A xenograft tumor was developed in BALB/c nude mice to further examine the role of hsa_circ_0051908 *in vivo*. Results showed that the tumor volume (Figure 5(a)) and weight (Figure 5(b)) were notably decreased by intratumoral injection of hsa_circ_0051908 siRNA. Additionally, E-cadherin was upregulated, while vimentin and Snail were downregulated by intratumoral injection of hsa_circ_0051908 siRNA compared with those in the NC group (Figure 5(c)). Immunohistochemical (IHC) staining confirmed that E-cadherin expression was higher, while vimentin and Snail expression was lower in the siRNA group than in the NC group (Figure 5(d)). In addition, IHC staining showed that the expression of Ki-67 was significantly inhibited by hsa_circ_0051908 siRNA compared with that in the NC group (Figure 5(d)). These results indicated that hsa_circ_0051908 promoted tumor growth and inhibited EMT *in vivo*. All data demonstrated that hsa_circ_0051908 promoted the progression of HCC.

## 4. Discussion

Clinical research and application of targeted and immunotherapy have enabled some patients with advanced HCC to receive surgical resection during translational therapy. However, delayed diagnosis and ineffective treatment regimens lead to poor prognosis with decreased overall survival in patients with HCC [18, 19]. Therefore, there is an urgent need to explore diagnostic biomarkers for HCC. In the present study, we found that hsa_circ_0051908 expression was increased in both HCC tissues and cell lines. We further found that hsa_circ_0051908 silencing significantly inhibited tumor growth by regulating the EMT process both *in vivo* and *in vitro*.

Increasing evidence indicates that abnormal expression of circRNAs affects the biological characteristics of liver cancer [8, 20]. For example, circPIP5K1A activates the PI3K-Akt signaling pathway through sponge adsorption of miR-671-5P, thereby promoting the progression of gastric cancer [21]; the circRNA circDLC1 inhibits MMP1-mediated liver cancer progression via interaction with HuR [22]; and exosomal circRNA-100338 promotes HCC metastasis via enhancing invasion and angiogenesis [23]. In the current study, hsa_circ_0051908 was upregulated in HCC tissues, suggesting that it plays an important role in HCC development. To date, the role of hsa_circ_0051908 in liver cancer has not been reported. Here, we showed that the proliferation, migration, and invasion of HCC cells were greatly decreased after hsa_circ_0051908 knockdown, while cell apoptosis was largely increased *in vitro*. Hsa_circ_0051908 silencing significantly inhibited tumor growth *in vivo*, suggesting that hsa_circ_0051908 can promote HCC progression.

The EMT process is crucial for tumor cells to acquire mobility, and it is induced by decreased expression of the epithelial-specific marker E-cadherin and increased expression of the mesenchymal markers N-cadherin and vimentin [24, 25]. Here, upregulated E-cadherin and downregulated vimentin after hsa_circ_0051908 silencing in HCC cells confirmed that hsa_circ_0051908 acts as an oncogene by mediating the EMT process. Snail affects tumor cell metastasis and invasion by participating in the regulation of EMT [26]. Snail can bind to the E-box of the E-cadherin gene promoter region to decrease E-cadherin protein expression, thus inducing EMT [26, 27]. Here, Snail expression was notably decreased after hsa_circ_0051908 silencing, indicating that hsa_circ_0051908 regulated HCC progression via regulating the EMT process.

This study has some limitations. In the present study, only the cancer-promoting effect of hsa_circ_0051908 was confirmed *in vitro* and *in vivo*. The molecular mechanism of hsa_circ_0051908 in HCC remains unclear. Subsequently, we will aim to elucidate the action mechanism of hsa_circ_0051908 in HCC progression using bioinformatics analysis and double luciferase and rescue experiments. Our future study will provide a sufficient theoretical basis for the application of hsa_circ_0051908 in liver cancer treatment.

In conclusion, our data indicated that hsa_circ_0051908 is an important regulatory molecule in the development and progression of liver cancer, and the circRNA is expected to be a prognostic indicator and therapeutic target for liver cancer.

## Figures and Tables

**Table 1:** The correlation relationship between hsa_circ_0051908 expression and clinical data.

Characteristics	Has-circ-0051908 expression		*P* value
	Low	High	
Gender	—	—	0.229
Male	20	14	—
Female	16	20	—
Tumor size (cm)	—	—	0.009^*∗∗*^
>3	11	21	—
≤3	25	13	—
Age	—	—	0.151
>60	15	20	—
≤60	21	14	—
Differentiation	—	—	0.001^*∗∗∗*^
High	10	25	—
Low/moderate	26	9	—
TNM	—	—	0.001^*∗∗∗*^
I–II	—	—	—
III–IV	—	—	—
Lymph node metastasis	—	—	0.004^*∗∗*^
Yes	12	23	—
No	24	11	—

^*∗∗*^*P* < 0.01;  ^*∗∗∗*^*P*  < 0.001.

## Data Availability

The data that supported the findings of this study are available upon reasonable request from the corresponding author.
